# Influence of an Esports Program on Problematic Gaming in Children with Autistic Spectrum Disorder: A Pilot Study

**DOI:** 10.3390/bs12060172

**Published:** 2022-05-31

**Authors:** Kentaro Kawabe, Fumie Horiuchi, Rie Hosokawa, Kiwamu Nakachi, Junya Soga, Shu-ichi Ueno

**Affiliations:** 1 Department of Neuropsychiatry, Ehime University Graduate School of Medicine, Toon 791-0295, Japan; kawabe.kentaro.fj@ehime-u.ac.jp (K.K.); dcoca14tf@gmail.com (R.H.); waffle9315@yahoo.co.jp (K.N.); s.erigeron@gmail.com (J.S.); ueno@m.ehime-u.ac.jp (S.-i.U.); 2 Center for Child Health, Behavior and Development, Ehime University Hospital, Toon 791-0295, Japan

**Keywords:** Esports, autism spectrum disorder, gaming disorder, problematic gaming, video games, mental health

## Abstract

Esports (electronic sports) programs are a variant of competitive gaming and have expanded worldwide in recent years. The prevalence of problematic gaming and gaming disorders (GD) is predicted to increase in adolescents. Children with autism spectrum disorder (ASD) have a high rate of digital gaming use, and their characteristics, such as social communication deficits and restricted interests, might contribute to problematic gaming. In this study, we aimed to examine whether participation in an Esports program would lead to problematic gaming or GD in children with ASD. The Internet Gaming Disorder Test (IGD-20) scores, Kid-KINDL scores, and gaming time at home were assessed in eight children with ASD before beginning the Esports program and at the three-month follow-up timepoint. The program was held once a week at the welfare service center, where the participants played a set game for 60 min. The results indicated there was no significant worsening in any of the scores after the program. Our program provided the participation time and frequency of Esports, type of game, and motivation of the participants are adequately considered. Even though this pilot study is limited by the small sample size, we concluded that the risk of these activities leading to problematic gaming might be low.

## 1. Introduction

**Figure 1:**
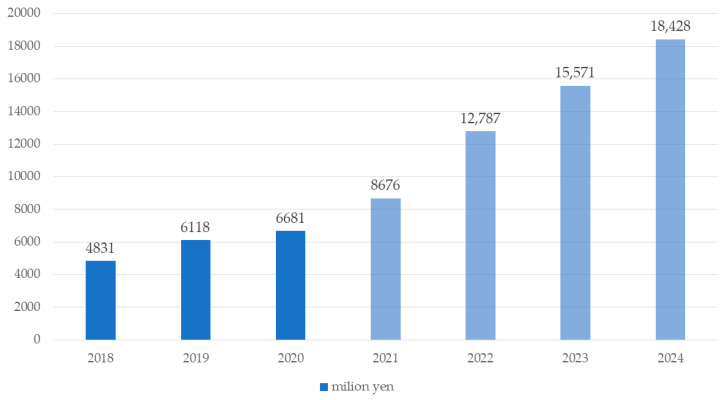
Domestic Esports market in Japan.

In recent years, there has been an exponential increase in the popularity of online video games, owing to technological advancements in network capabilities. Online video games range from strategic maze-based games and falling blocks games where players battle, shooters games, and sports games where a player may team up to play with a small group of players, and to large scale multiplayer online battle, and so on [1,2]. The term Esports, is short for electronic sports, and Esports are considered a variant of video gaming. While there is no consensus on the definition, it is generally agreed that Esports are competitive gaming activities [3]. Over millions of players play games and participate in electronic competitions daily, leading to a rapid proliferation in the number of players globally. Esports is growing around the world, where more and more individuals are engaging as players or spectators [4]. The most attractive attention point of Esports in Japan is the element of teamwork. Esports in Japan had not flourished despite its large video game market; there were various Esports tournaments that have started in recent years. In addition, the official Esports organization, which is called the Japan Esports Union (JeSU) was established in 2017. Junior and high schools are adopting Esports at an increasing pace, the National Athletic Meet cultural program “Prefectural Esports Tournament” being held per year since 2019 in Japan [5]. In addition, various Esports tournaments are being held for high school students, such as ALL JAPAN High School Esports Championship [6], and the National High School Esports Tournament [7]. This rapid global growth of Esports has led not only to an increased number of players and competitors, but also creating new labor, political economy, employment, and marketing [8]. According to ewzoo’s Global Esports & Live Streaming Market Report, global Esports revenues will grow to $1084 million in 2021, a year-on-year growth of +14.5%, up from $947.1 million in 2020 [9]. As shown in Figure 1, the size of the domestic E-sports market in 2020 reached 6.68 billion yen (about $52.3 million), 109% of the previous year’s level in Japan (after 2021 are forecasts) [10].

In the medical and welfare fields, various approaches are focusing on the theme of Esports and disabilities [11]. To utilize as para Esports, a new gaming controller has been developed for persons with upper limb deficiencies [12]. A notable point, Esports has the potential to enable patients with physical or mental disabilities to play games competitively alongside youths without these disabilities. In addition, Esports have also had a positive effect on the physical fitness in children with intellectual disabilities [13]. However, higher engagement with gaming was related to physical activity, nutrition, physical lifestyle, and sleep quality [3]. In addition, the prevalence of problematic gaming and gaming disorders (GD) are predicted to increase due to Esports popularity [14]. GD are characterized by impaired control over gaming activities, increasing levels of priority given to gaming instead of other activities to the extent that gaming takes precedence over other interests and daily activities, and continuation or escalation in gaming activities despite negative consequences [15]. The term “problematic gaming” refers to the gaming habits of individuals who may be at risk of GD, which is usually determined by the individual exhibiting some but not all the symptoms of GD [16].

A recent systematic review has reported that problematic gaming is highly prevalent in boys and young men with autism spectrum disorder (ASD) [17]. According to the Diagnostic and Statistical Manual of Mental Disorders, Fifth Edition (DSM-5), criteria (American Psychiatric Association 2013), ASD has the following clinical features that might contribute to problematic media use and gaming: social communication deficits, restricted interests, sensory differences, and executive functioning weaknesses. ASD have been increasing and the median prevalence of ASD was reported to be 3.32% in Japan [18]. Environmental toxins and stressors, mitochondrial dysfunction, impaired immune responses, and dysregulation of the tryptophan (TRP) catabolite pathway are involved in the pathogenesis of ASD [19,20]. The TRP-kynurenine metabolic pathway is the main catabolic route of TRP metabolism, which has been associated with a wide range of diseases including cancer, autoimmune diseases, inflammatory diseases, neurologic diseases, and psychiatric disorders [21]. Inflammation and immune signaling dysregulation can strongly influence neuropsychiatric behavior beyond just ASD, with evidence pointing toward roles in bipolar disorder and PTSD, and perhaps most emphatically in schizophrenia [22].

Craig et al. [23] reported that social difficulties that are faced in real life might be reflected in problematic gaming among individuals with ASD. A recent study demonstrated that boys with ASD played video games for longer periods, played alone rather than in the company of others, and played less frequently in multiplayer mode [24]. Thus, there is reason to suspect that children with ASD might be at a greater risk of problematic gaming if they get involved in an Esports program. Therefore, the aim of the current study was to examine the influence of Esports programs on problematic gaming in children with ASD.

## 2. Materials and Methods

### 2.1. Participants and Design

The study participants were required to meet the following inclusion criteria in this study. The inclusion criteria were: (1) children aged 6–12 years; (2) those diagnosed with ASD; (3) those who were attending the welfare service center for neurodevelopmental disorders, mainly during after-school day care, in Ehime prefecture; and (4) those who signed informed consent forms along with their parents. The exclusion criteria were: (1) categorized as having GD according to the IGD-20 Test, (2) having an intellectual disability, (3) having a psychiatric disorder, and (4) those refusing to provide informed consent. Diagnosis was based on the criteria for ASD as defined in the DSM-5; Autism Diagnostic Observation Schedule, Second Edition (ADOS-2); and Parent-Interview ASD Rating Scale-Text Revision (PARS-TR) [25]. The study period was from April 2020 to August 2020.

### 2.2. Procedure

The participants were instructed to take part in an Esports program as part of after-school activities. The program was held for 60 min once a week at the welfare service center. The games that were used in this study were titled “Puyo Puyo.” (https://puyo.sega.com/champions/ (accessed on 1 May 2022)) and “Super Bomberman R Online.” (https://www.konami.com/games/bomberman/online/us/en/ (accessed on 1 May 2022)). PuyoPyo is a license certification title as Esports by JeSU. Super Bomberman R is an action-maze game that was developed in 2017. Part of the Bomberman franchise, it is the sixth installment of the Super Bomberman series and the first game in the series to be released in 20 years. An online-only version titled Super Bomberman R Online was released in 2020, and often played by various Esports tournaments in Japan. The Esports program was conducted throughout the year. The participants were evaluated using self-reported and parent-reported questionnaires before beginning the Esports program (pre) and 3 months after the program (post).

### 2.3. Instruments

#### Internet Gaming Disorder Test (IGD-20 Test)

The IGD-20 Test is a 20-item parent-reported questionnaire that is used to assess IGD symptoms. It assesses the symptoms of IGD across six subscales (salience, mood modification, tolerance, withdrawal symptoms, relapse, and conflict). All the subscales comprise of three items, apart from the conflict subscale, which has five items. The answers are scored on a Likert scale ranging from 1 (strongly disagree) to 5 (strongly agree). The minimum and maximum scores are 20 and 100, respectively, and the participants who scored 71 or more were classified as having IGD [26]. The IGD-20 Test has been psychometrically validated and culturally adapted for Spanish, Arabic, and Korean speakers. In the present study’s sample, the Cronbach’s alpha coefficient was 0.906.

The KINDL was developed to measure generic health-related quality of life (QOL) in children aged 4 to 17 years using both a child-reported questionnaire and a parent-observer version [27]. The questionnaire consists of 24 items addressing six subscales: physical well-being, emotional well-being, self-esteem, family, friends, and school life. There are three versions of the KINDL that are available: Kiddy-KINDL for children aged 4–6 years, Kid-KINDL for children aged 7–13 years, and Kiddo-KINDL for children aged 14–17 years. Kid-KINDL is able to measure degrees of health and adaptability in relation to QOL in children [28]. In this study, we used self-reported versions of the Kid-KINDL. The raw scores were calculated for each of the six subscales and were transformed into a final score on a scale of 0–100 to facilitate interpretation. The Kid-KINDL reported acceptable reliability and validity [29], and test-retest reliability also was acceptable in all the subscales and the total score [30]. In the present study’s sample, the Cronbach’s alpha coefficient was 0.636.

### 2.4. Statistical Analyses

The data are expressed as the mean and standard deviation (SD). Internal consistencies within individual subscales were evaluated using Cronbach’s alpha. Differences between the pre- and post-study parameters were compared using the Wilcoxon signed-rank test. All statistical analyses were conducted using SPSS for Windows, version 24 (IBM Corp., Armonk, NY, USA). The level of statistical significance was set at *p* < 0.05.

## 3. Results

The study participants included eight children (all males) between 6 and 12 years of age (mean age, 8.8 years; SD, 2.3). The individual results are shown in Table 1. Among these participants, four boys had ASD and four boys had ASD and attention deficit hyperactivity disorder (ADHD).

**Figure 2:**
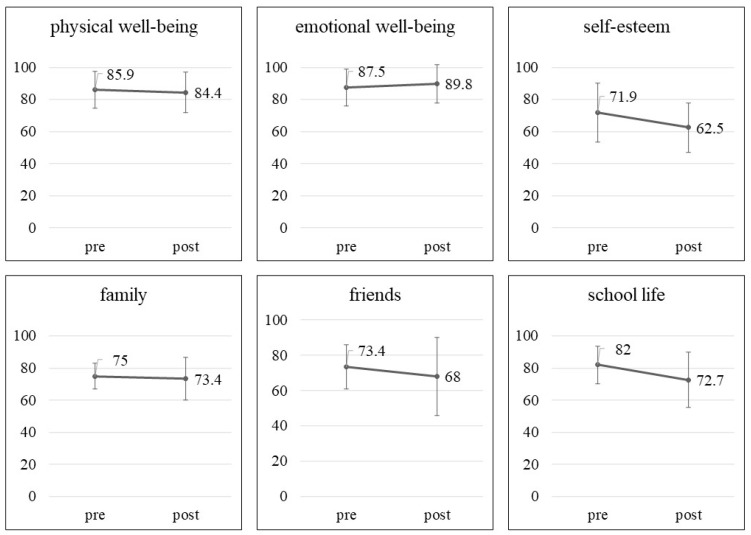
Changes in the Kid-KINDL subscale scores between baseline and 3 months after starting the Esports program in children with autism spectrum disorder. Wilcoxon signed-rank test: physical well-being, *p* = 0.87; emotional well-being, *p* = 0.56; self-esteem, *p* = 0.14; family, *p* = 0.66; friends, *p* = 0.77; school life, *p* = 0.17.

The duration of gaming on weekdays was 12.5 ± 21.9 min at the pre-study timepoint and 21.3 ± 41.5 min at the post-study timepoint (*p* = 0.18). During the weekend, the participants played games for 88.8 ± 50.0 min at the pre-study timepoint and for 116.9 ± 108.7 min at the post-study timepoint (*p* = 0.47). The IGD-20 Test scores were not significantly different between the pre- (40.4 ± 7.7) and post-study (39.8 ± 12.1) timepoints (*p* = 0.73). Figure 2 presents the changes in the mean scores of the Kid-KINDL subscales, which were not significantly different between the pre- and post-study timepoints. In brief, there were no significant changes in the duration of gaming at home, IGD-20 scores, and Kid-KINDL subscale scores between the pre- and post-study timepoints. 

## 4. Discussion

The present study provides important evidence that can aid in elucidating the relationship between Esports activities and problematic gaming in boys with ASD. More specifically, the study assessed the influence of Esports on problematic gaming symptoms and gaming time as well as their effect on the QOL in boys with ASD. None of the scores that were evaluated changed significantly after the program. 

The content, time, and frequency of the Esports activities were decided before the implementation of the program in this study. Previous studies and reviews have reported that certain problematic issues that are related to gaming, such as aggressive behavior, mental state changes, and gaming addiction, were found in younger children and adolescents [31]. Video games with violent content cause aggressive behavior in players, especially in younger children and adolescents [32]. Younger children with ASD may not have the emotional maturity to understand violent gaming content. Although many researchers and reports that attempt to explain the links between video gaming and its adverse effects are based on a limited understanding of the background factors. Hartant et al. suggested that the social context, type, motivation, time and day, and the amount of video gaming activities are adequately considered [33]. For this study, we chose competitive puzzle games, played one hour per week, and the day is a weekday. In Japan, Computer Entertainment Rating Organization (CERO) which have game rating system, was established in 2003. Although the CERO game rating modeled itself after the United States’ regulatory board the ESRB, it was regulated by the Japanese government. CERO is displayed on video game packaging icons depicting content such as violence, alcohol use, sexual content, etc. CERO also has ratings for age appropriateness: A for all ages, B for over 12 years old, C for over 15 years old, D for over 17 years old, and Z for over 18 years old. Therefore, we selected the CERO rating “A” games without contain violence, such as killing enemies or players. Furthermore, coaches in the welfare center were assigned to instruct the participants in this study. Guidance regarding the desirable usage of video games might be needed for children with ASD. Similar to the findings in our study, previous studies have reported significantly longer times and higher rates of digital media use in children with ASD than in typically developing children [34,35]. In a systematic review, long hours of online gaming were found to be associated with psychological problems, such as depression, social phobia, obsession-compulsion, interpersonal sensitivity, hostility, phobic anxiety, paranoid ideation, psychoticism, ADHD, and gaming addiction [36]. The time spent playing is a key variable in problematic gaming; thus, the present study was designed such that the time that was allotted to the Esports activity would be relatively short [37]. Future research is needed to determine the psychological effects of gaming duration.

This study was conducted in children with ASD. Although the clinical features of ASD include social communication deficits, the included children found the games easy to understand and play because the programs utilize various ways of communication, including both oral and written communication. In-game informational and instrumental support lead to emotional and esteem support [38]. Moreover, they received instructions from coaches before and during the playing time. For a supporter, game strategy training will be a good platform to observe and understand the communication patterns and positionings in a team [39]. Sundberg [40] reported that online games might help individuals with ASD in building and sustaining friendships and in reducing feelings of loneliness. Therefore, Esports could be used as an effective tool for managing the ASD-related characteristics in children.

This study had several limitations. First, the sample size was small and a part of participants had a combined ASD and ADHD diagnosis. In addition, all the participants were boys, so a selection bias could not be ruled out. Our power calculations were based on a restricted number of available participants data; this study is just for your reference. Second, our study could not control for confounders that were related to time due to the use of a pre-post intervention study design without a control group. Third, our assessment relied on self and parent reports. Forth, we did not control for confounding factors, such as the domestic environment, including economic status.

Research on digital media use in children, especially in those with ASD, commonly focuses on physical and mental health outcomes and pathological use [41]. Even though this study is limited by the small sample size with an extreme risk of sample bias that is related to the selected patient population from the same gender and same setting, our findings provide the necessary framework to the attended Esports program in ASD. In the future, Esports programs will lead to changes in communicative adjustment in children with ASD.

## Figures and Tables

**Table 1:** Change in the data that were derived from the evaluation instruments after the Esports program.

No.	Age, Years	Sex	Diagnosis	Duration of Gaming at Home (Weekday, min)		Duration of Gaming at Home (Weekend, min)		IGD-20		Kid-KINDL											
										Physical Well-Being		Emotional Well-Being		Self-Esteem		Family		Friends		School Life	
				pre	post	pre	post	pre	post	pre	post	pre	post	pre	post	pre	post	pre	post	pre	post
1	7	Boy	ASD	0	0	120	120	43	39	87.5	100	62.5	68.8	62.5	62.5	81.3	68.8	68.8	75	62.5	75
2	8	Boy	ASD	0	0	90	120	42	60	100	68.8	87.5	93.8	75	93.8	81.3	68.8	93.8	87.5	87.5	87.5
3	9	Boy	ASD	0	0	120	60	39	27	81.3	87.5	87.5	100	93.8	62.5	87.5	75	81.3	87.5	87.5	81.3
4	12	Boy	ASD	10	20	20	35	36	24	75	62.5	100	93.8	87.5	62.5	68.8	100	87.5	25	87.5	75
5	6	Boy	ASD/ ADHD	60	120	120	360	44	48	100	87.5	100	87.5	87.5	56.3	75	62.5	62.5	68.8	100	68.8
6	7	Boy	ASD/ ADHD	30	30	120	120	48	47	81.3	87.5	87.5	100	62.5	62.5	62.5	75	62.5	87.5	81.3	62.5
7	9	Boy	ASD/ ADHD	0	0	0	0	24	31	68.8	87.5	87.5	100	37.5	37.5	75	56.3	68.8	62.5	81.3	93.8
8	12	Boy	ASD/ ADHD	0	0	120	120	47	42	93.8	93.8	87.5	75	68.8	62.5	68.8	81.3	62.5	50	68.8	37.5

Abbreviations: IGD-20, Internet Gaming Disorder Test; ASD, autism spectrum disorder; ADHD, attention deficit hyperactivity disorder. pre: before beginning the Esports program, post: 3 months after the program.

## Data Availability

Not applicable.
